# Variations in and predictors of the occurrence of depressive symptoms and mood symptoms in multiple sclerosis: a longitudinal two-year study

**DOI:** 10.1186/s12883-016-0551-1

**Published:** 2016-03-05

**Authors:** Sverker Johansson, Kristina Gottberg, Marie Kierkegaard, Charlotte Ytterberg

**Affiliations:** Department of Neurobiology, Care Sciences and Society, Karolinska Institutet, SE-141 83 Huddinge, Sweden; Department of Clinical Neuroscience, Karolinska Institutet, Stockholm, Sweden; Department of Physiotherapy, Karolinska University Hospital, Stockholm, Sweden

**Keywords:** Depression, Longitudinal, Mood, Multiple sclerosis, Predictors

## Abstract

**Background:**

There is limited knowledge regarding how depressive symptoms and a cluster of specific mood symptoms in people with multiple sclerosis (MS) vary over time and how they are influenced by contributing factors. Therefore, the aims of this study were a) to describe variations over 2 years in the occurrence of depressive symptoms and mood symptoms in a sample of people with MS, and b) to investigate the predictive value of sex, age, coping capacity, work status, disease severity, disease course, fatigue, cognition, frequency of social/lifestyle activities, and perceived impact of MS on health, on the occurrence of depressive symptoms and mood symptoms.

**Methods:**

Through using a protocol of measures of functioning and perceived impact of MS on health, comprising of the Beck Depression Inventory, 219 people with MS were assessed at 0, 12 and 24 months. Predictive values were explored with Generalised Estimating Equations.

**Results:**

Proportions with depressive symptoms varied significantly (*p* < 0.001) from 21 to 30 % between the three time points. Proportions with mood symptoms varied significantly (*p* < 0.001) from 14 to 17 % between the three time points. Weak coping capacity and reduced frequency of social/lifestyle activities predicted the occurrence of depressive symptoms and mood symptoms, as did the psychological impact of MS on health in interaction with time. For people with MS of working age, not working predicted the occurrence of depressive symptoms and mood symptoms, as did the physical impact of MS on health on the occurrence of mood symptoms.

**Conclusions:**

The occurrence of depressive symptoms and mood symptoms in people with MS vary over a 2-year time period; almost half have depressive symptoms at least once. Health care services should develop strategies aimed at identifying people with MS who are depressed or who develop depressive symptoms. Interventions for alleviating depressive symptoms should consider the individual’s coping capacity and perceived impact of MS on health, and facilitate their ability to maintain participation in valued everyday activities.

## Background

Multiple sclerosis (MS) is a chronic, progressive disease in the central nervous system with an unpredictable course. MS often appears in early adulthood, and for most afflicted people with MS (PwMS) will cause a mixture of disabilities that vary and might progress over time [1], thereby implying uncertainty over many years. The combination of unpredictability and variability of disabilities as well as the coping capacity of PwMS have been proposed as factors influencing quality of life [2, 3]. The uncertainty of living with MS is a psychosocial factor that might also influence mood [4]. The prevalence of depressive symptoms in PwMS, ranging from 26 to 42 % [5–7], is higher compared to the prevalence in the general population [7] and in people living with other chronic conditions [7]. Most longitudinal studies of depressive symptoms in PwMS report no change over time [8–11], but a change has been reported regarding mood symptoms [8], a specific cluster of depressive symptoms considered not to overlap with MS-related symptoms such as various impairments related to physical or cognitive functioning [12]. This was confirmed in our previous study of a cohort of PwMS who were followed over a period of 2 years in which the scores of depressive symptoms did not change whereas scores of specific mood symptoms (feeling sad; discouraged about the future; dissatisfied and bored; crying; irritability; and loss of interest) did [13].

One limitation in studies of depression in MS is that the instruments used describe the global indices of depression and do not differentiate diverse depressive symptom clusters such as mood symptoms from somatic and cognitive/evaluative symptoms which may overlap MS-related symptoms [8]. Studying mood symptoms is important since it may inform health services about which interventions should be used for alleviating mood symptoms and which should be used for alleviating other consequences from MS.

Depressive symptoms in PwMS are associated with fatigue [14], cognitive impairment [15], reduced frequency of social/lifestyle activities [16], and limitations in working ability [17, 18]. Associations with weak capacity to cope with and adjust to stressors in life [6] such as living with MS, and with reduced quality of life [19, 20] have also been reported. The results regarding associations between depressive symptoms and sex and age are more varied [6, 21], as are the associations with disease severity [5, 6]. In addition, associations between depressive symptoms in PwMS and mortality [22] and death by suicide [23] have been reported. However, associations between mood symptoms and other disabilities such as fatigue and cognitive impairment have not been explored.

There is limited knowledge about how depressive symptoms and the cluster of mood symptoms which are not overlapped by MS-related symptoms vary and are influenced by different factors over time. Even if evidence-based principles for treatment of depressive symptoms are now available, such as pharmacological [24] and psychological treatments [25, 26], there is a need to identify factors that are associated with depressive symptoms as well as with mood symptoms to possibly decrease the impact of these symptoms. Therefore, the aims of this 2-year study on PwMS were:

- to describe variations in the occurrence of depressive symptoms and mood symptoms; and
- to investigate the predictive value of selected factors – sex, age, coping capacity, work status, disease severity, disease course, fatigue, cognitive function, frequency of social/lifestyle activities, and perceived physical and psychological impact of MS on health – on the occurrence of depressive symptoms and mood symptoms, respectively.

## Methods

### Participants and procedures

Data were collected in a prospective, observational study over 2 years from a cohort of PwMS in which functioning and perceived impact of MS on health were explored. Eligibility for inclusion included those PwMS (*n* = 255) scheduled for an outpatient visit from 1 February 2002 to 12 June 2002 with either of two senior neurologists at the MS Centre of the Department of Neurology at Karolinska University Hospital Huddinge in Stockholm, Sweden. Altogether 219 PwMS agreed to participate and were consecutively included in the study after informed consent had been obtained. Follow-ups were performed every 6 months (at baseline and at 6, 12, 18 and 24 months) in connection with regular visits by the PwMS to her/his senior neurologist who determined disease severity and disease course. The remaining data were collected by investigators, one of five research physiotherapists, and when possible by the same investigator and at the same time of day for all time points. Detailed descriptions of demographic and disease-related data in the sample at baseline have been reported elsewhere [27]. The Regional Ethical Review Board in Stockholm approved the study, Dnr 449/01.

### Instruments

Data used in the study were collected with a range of standardised and validated instruments or with structured interviews.

Depressive symptoms were assessed with the Beck Depression Inventory (henceforth referred to as BDI Total) [28] at baseline, 12 and 24 months. The BDI Total consists of 21 items related to depression, each of which is self-rated from 0 (absent) to 2 or 3 (severe); the scale ranges from 0 to 62 points. At each assessment the PwMS were categorised as having depressive symptoms if they had a BDI score of 13 or higher [18]. The 21 BDI items were also grouped into three clusters of depressive symptoms dealing with mood, evaluative/cognitive and somatic/vegetative symptoms [12]. The six items included in the mood cluster (henceforth referred to as BDI Mood) were the following: feeling sad; discouraged about the future; dissatisfied and bored; crying; irritability; and loss of interest. The cluster ranges from 0 to 18 points and was dichotomised using a cut-off score derived from the overall cut-off score for detecting depressive symptoms in MS [18]. Thus, PwMS were categorised as having mood symptoms if their score was five or above on the BDI Mood.

Data regarding sex, age and work status were collected by interview at baseline, as were data regarding current use of anti-depressant drugs, psychological treatment and immunomodulatory pharmacological treatment. To assess coping capacity the 13-item version of the Sense of Coherence (SOC) scale was used [29, 30]. SOC indicates an individual’s capacity to cope with stressful life events. Disease severity was assessed with the Expanded Disability Status Scale (EDSS) [31]. Information about time since diagnosis was collected from the medical records or, when unregistered, by interview. Fatigue was assessed with the Fatigue Severity Scale (FSS) [32] reflecting the severity of fatigue and its impact on daily functioning. Cognitive function was assessed with the Symbol Digit Modalities Test (SDMT) [33]. The SDMT assesses the capacity to direct attention quickly and accurately and was primarily administrated with written reply. However, for those PwMS unable to write an oral reply was used. To assess frequency of social/lifestyle activities the Frenchay Activities Index (FAI) was used [34, 35]. The FAI consists of 15 items covering domestic chores and outdoor, leisure and work activities that require initiative and organisation on the part of the individual. The perceived physical and psychological impact of MS on health was assessed with the disease-specific Multiple Sclerosis Impact Scale (MSIS-29) [36]. The MSIS-29 consists of one physical subscale with 20 items and one psychological subscale with nine items.

Data regarding the selected factors used as independent variables in the study were collected at baseline, except coping capacity, which was collected at 6 or 12 months.

### Statistical analysis

Descriptive statistics were used to present depressive symptoms and mood symptoms in the sample. Criteria for categorisation of the characteristics used as independent variables in the study are presented in Table 1. A chi-squared test was used in order to analyse the differences between the three time points in proportions of the BDI Total and BDI Mood, respectively. A chi-squared test was also employed for univariate analyses of differences with regard to the independent variables between PwMS with depressive symptoms versus no depressive symptoms and PwMS with mood symptoms versus no mood symptoms. A probability (p) value ≤ 0.05 was considered statistically significant.

**Table 1:** Independent variables, categorisation criteria and characteristics of 199 PwMS completing the BDI at one or several time points

Independent variables and methods used	Categorisation criteria		Characteristics of the sample, *n* (%)
Sex	Female		135 (68)
	Male		64 (32)
Age^a^	<47 years		102 (51)
	≥47 years		97 (49)
Coping capacity *Sense of Coherence scale* ^b^	Weak, < 55		23 (12)
	Moderate/Strong, ≥ 55		176 (88)
Work status^c^ *Interview*	Working, full- or part-time		115 (58)
	Not working		68 (34)
	Retired		16 (8)
Disease severity *Expanded Disability Status Scale* (EDSS)^d^	EDSS mild	0.0–3.5	125 (63)
	EDSS moderate	4.0–5.5	35 (17.5)
	EDSS severe	6.0–9.5	39 (19.5)
Disease course	Relapsing remitting course		122 (61)
	Primary or secondary progressive course		77 (39)
Fatigue *Fatigue Severity Scale*	Non-fatigue	≤4.0	67 (34)
	Borderline fatigue	4.0 < FSS < 5.0	32 (16)
	Fatigue	≥5.0	100 (50)
Cognitive function *Symbol Digit Modalities Test* ^e^	No impairment		104 (52)
	Impairment		95 (48)
Frequency of social/lifestyle activities *Frenchay Activities Index* ^f^	Normal		111 (56)
	Reduced		88 (44)
Perceived physical impact of MS *Multiple Sclerosis Impact Scale* ^g^	Small impact		100 (50)
	Large impact		99 (50)
Perceived psychological impact of MS *Multiple Sclerosis Impact Scale* ^g^	Small impact		92 (46)
	Large impact		107 (54)

^a^The mean age of the sample at baseline

^b^Sex-related norms [30]

^c^Work status: applied if < 65 years of age, the customary retirement age in Sweden, n = 183

^d^The categorisation applied in the Swedish MS Registry

^e^Age-related norms [33], written or oral reply, −1.5 SD [33]

^f^Age- and sex-related norms [34], < lower quartile at baseline

^g^Categorised according to the median of its distribution in the sample at baseline

Four models using Generalised Estimating Equations (GEE) employing proportional odds were used to explore the predictive value of the independent variables on two different dependent variables: the occurrence of depressive symptoms (as categorised with the BDI Total) and the occurrence of mood symptoms (as categorised with the BDI Mood). The BDI Total was the dependent variable in Model 1 and Model 2, and the BDI Mood was the dependent variable in Model 3 and Model 4. The dependent variables comprised data from the three time points when they were assessed (at 0, 12 and 24 months). Independent variables at baseline were included in the models together with the time factor (0, 12 and 24 months). The independent variables were sex, age, coping capacity, work status, disease severity, disease course, fatigue, cognitive function, frequency of social/lifestyle activities, perceived physical as well as psychological impact of MS on health, and time. Model 1 and Model 3 contained all independent variables except work status. Model 2 and Model 4 contained all independent variables and PwMS < 65 years of age, the customary age for retirement in Sweden. Using GEEs allowed for the inclusion of participants with data collected on the BDI Total or on the BDI Mood at least at one time point and complete data for the independent variables. Interactions between time and the independent variables were controlled for, as were interactions between coping capacity and fatigue, disease severity and disease course, and between disease severity and the perceived physical and psychological impact of MS, respectively. Stepwise backward selection was employed using probability values of ≥ 0.05 for variable removal. Pair-wise comparisons were adjusted for multiple comparisons with the Bonferroni correction. The predictive values are presented as odds ratios (OR) with 95 % confidence intervals (CI) and probability values.

All statistical analyses were performed in IBM SPSS Statistics, version 20 or 22 (SPSS Inc., Chicago, Illinois, USA).

## Results

Of the 219 PwMS included, 200 completed the study; 7 died and 12 withdrew. At the various time points the BDI was completed by 94 to 96 % of participants. A total of 199 PwMS completed the BDI for at least one time point; characteristics of this sample at baseline are presented in Table 1. Of those, 57 PwMS (29 %) reported using anti-depressant drugs and 15 PwMS (8 %) reported having had contact with a psychologist over a shorter or longer period during the 2 years. A total of 154 PwMS (77 %) reported use of immunomodulatory pharmacological treatment over the 2 years.

A total of 185 PwMS completed the BDI at all three time points. In this group the proportion with depressive symptoms according to the BDI Total varied significantly (*p* < 0.001) from 21 to 30 % between the three time points. A total of 124 PwMS (67 %) remained in the same category at all three time points; 16 PwMS (9 %) had depressive symptoms and 108 (58 %) had no depressive symptoms. Sixty-one PwMS (33 %) changed BDI Total category: 22 PwMS (12 %) changed from no depressive to depressive symptoms at one time point, 23 PwMS (12 %) changed from depressive to no depressive symptoms at one time point, and 16 PwMS (9 %) changed category two times.

Among those PwMS who completed the BDI at all three time points (*n* = 185) the proportion of PwMS with mood symptoms according to the BDI Mood varied significantly (*p* < 0.001) from 14 to 17 % between the three time points. A total of 139 PwMS (75 %) remained in the same BDI Mood category at all three time points; 8 PwMS (4 %) had mood symptoms and 131 (71 %) had no mood symptoms. Forty-six PwMS (25 %) changed BDI Mood category: 19 PwMS (10 %) changed from no mood symptoms to mood symptoms at one time point, 17 (9 %) changed from mood symptoms to no mood symptoms at one time point, and 10 PwMS (5 %) changed BDI Mood category two times.

**Fig. 1:**
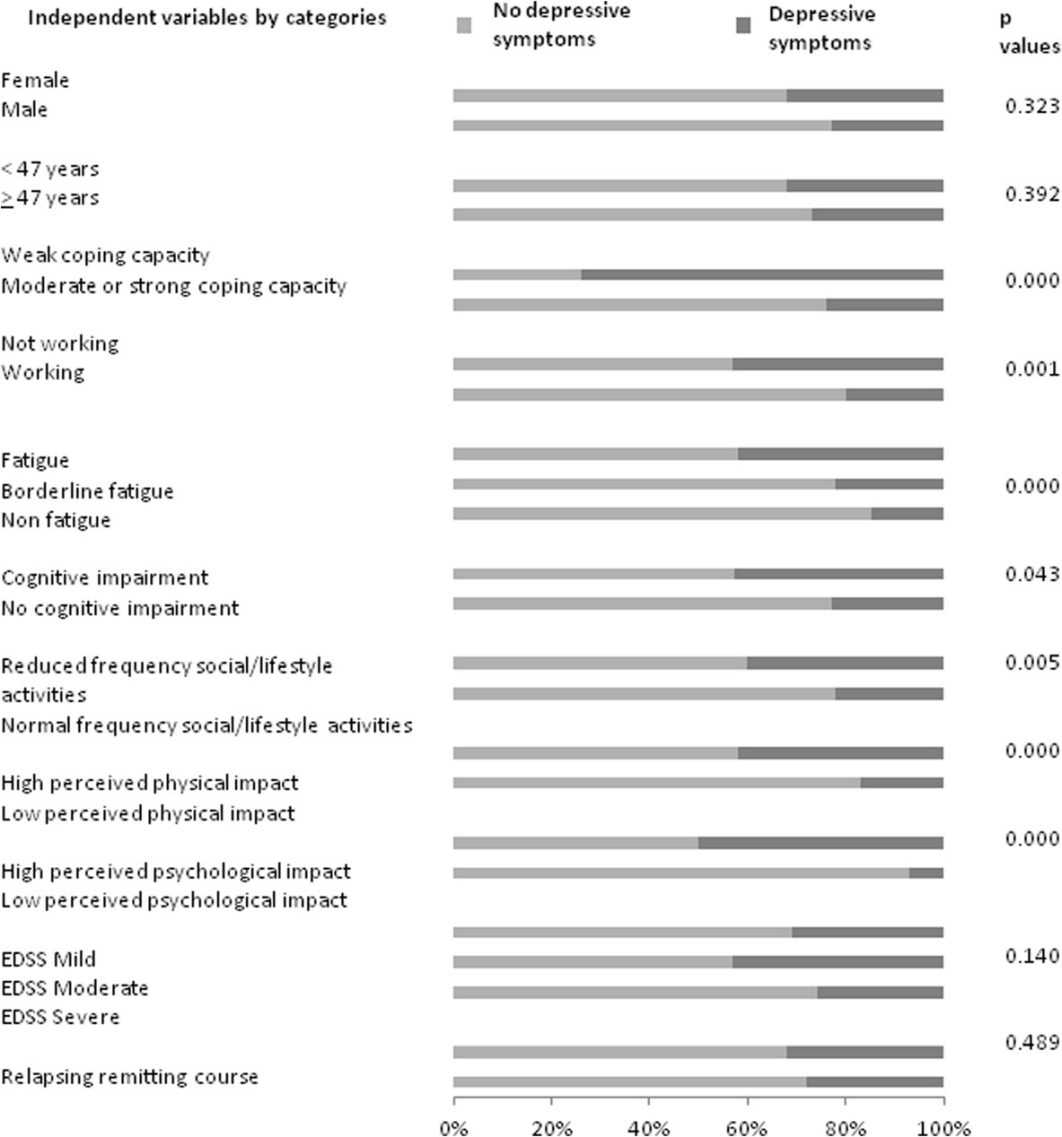
Proportions per category of independent variables by BDI Total category; p-values of univariate analyses (*n* = 199)

**Fig. 2:**
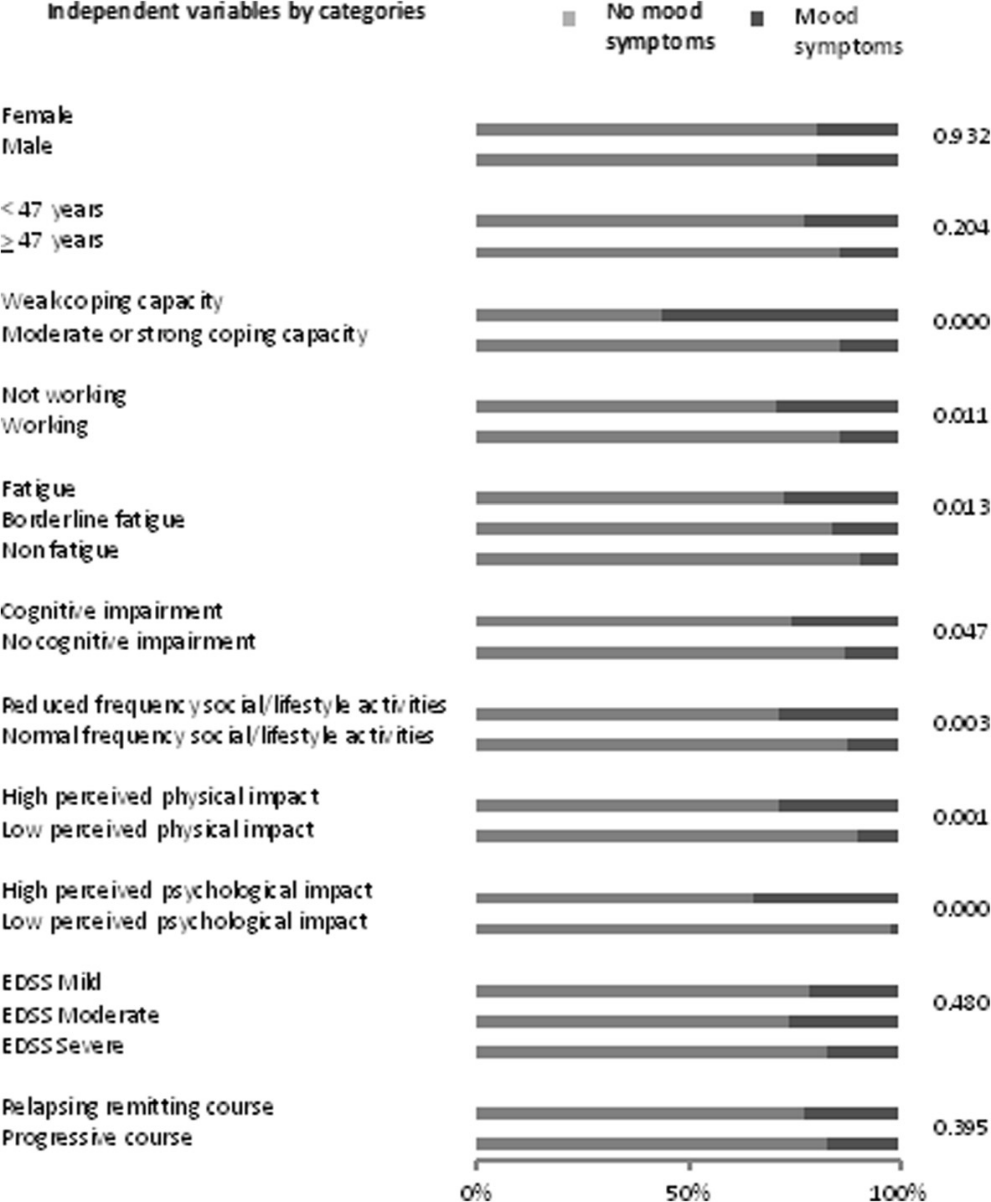
Proportions per category of independent variables by BDI Mood category; p-values of univariate analyses (*n* = 199)

Using GEEs allowed the inclusion of 199 PwMS with at least one BDI Total score or one BDI Mood score and complete data for the independent variables. Figure 1 presents the proportions per category of the independent variables by BDI Total and p values of univariate analyses at baseline. Figure 2 presents proportions per category of the independent variables by BDI Mood and p values of univariate analyses at baseline.

The probability of belonging to a certain category of the BDI Total or of the BDI Mood did not change significantly over time in the GEE models. The perceived psychological impact of MS on health interacted with time, i.e. the odds for depressive symptoms were higher at 12 and 24 months compared with baseline among PwMS with high psychological impact of MS. No other interactions were found.

The results of the GEE with the BDI Total as the dependent variable – Model 1 (*n* = 199) and Model 2 (PwMS < 65 years of age, *n* = 183) – are presented in Table 2. In Model 1 weak coping capacity and reduced frequency of social/lifestyle activities were independent predictors for the occurrence of depressive symptoms as was a high perceived psychological impact of MS in interaction with time. In Model 2 weak coping capacity and not working were independent predictors for the occurrence of depressive symptoms as was a high perceived psychological impact of MS in interaction with time.

**Table 2:** Factors predicting occurrence of depressive symptoms (Models 1, 2) and mood symptoms (Models 3, 4)

Dependent Variable	Independent variable categorisation	Model 1 and 3 OR (CI)^a^ *n* = 199^b^	P-value	Model 2 and 4 OR (CI) ^a^ *n* = 183^c^	*P*-value
Depressive symptoms^d^	Weak coping capacity Moderate/Strong	5.18 (2.64–10.10) 1	<0.001	4.90 (2.57–9.35) 1	<0.001
	Reduced frequency of social/lifestyle activities Normal frequency	2.20 (1.28–3.77) 1	0.004	Not significant in this group	
	Not working Working	Not applicable		2.50 (1.43–4.35) 1	0.001
	High psychological impact of MS/Time:				
	24 months Baseline	3.94 (1.25–12.35) 1	0.019	5.78 (1.61–20.83) 1	0.007
	12 months Baseline	3.34 (1.02–10.99) 1	0.047	3.89 (1.07–14.29) 1	0.040
Mood symptoms^e^	Weak coping capacity Moderate/Strong	6.06 (3.05–12.05) 1	<0.001	5.81 (3.08–11.11) 1	<0.001
	Reduced frequency of social/lifestyle activities Normal frequency	2.29 (1.25–4.22) 1	0.008	Not significant in this group	
	Not working Working	Not applicable		1.93 (1.04–3.60) 1	0.037
	High physical impact of MS Low impact	Not significant in this group		2.27 (1.02–5.05) 1	0.044
	High psychological impact of MS/Time:				
	24 months Baseline	5.85 (1.04–33.33) 1	0.046	6.37 (1.10–37.04) 1	0.038
	12 months Baseline	3.91 (0.83–18.52) 1	0.084	3.79 (0.80–17.86) 1	0.093

^a^ Estimated odds ratios (OR), 95 % confidence intervals (CI) and p values for the predictive value of the selected factors

^b^Model 1 and 3: *n* = 199 – all PwMS were included, these models included all the independent variables except work status

^c^Model 2 and 4: *n* = 183 – PwMS < 65 years of age were included, these models included all the independent variables

^d^BDI Total: The Beck Depression Inventory

^e^BDI Mood: The mood cluster in the Beck Depression Inventory

The results of the GEE with BDI Mood as the dependent variable – Model 3 (*n* = 199) and Model 4 (PwMS < 65 years of age, *n* = 183) – are presented in Table 2. In Model 3 the same variables as in Model 1 were independent predictors for occurrence of mood symptoms, i.e., weak coping capacity, reduced frequency of social/lifestyle activities, and a high perceived psychological impact of MS in interaction with time. In Model 4 the same variables as in Model 2 were independent predictors for occurrence of mood symptoms, i.e., weak coping capacity, not working, and a high perceived psychological impact of MS in interaction with time. Furthermore, in Model 4 a high perception of the physical impact of MS was also an independent predictor.

## Discussion

The present study showed that in a sample of PwMS the proportions with depressive symptoms as well as mood symptoms varied significantly over 2 years. Coping capacity and reduced frequency of social/lifestyle activities were independent predictors for the occurrence of depressive symptoms and mood symptoms, as was psychological impact of MS on health in interaction with time. Work status, but not reduced frequency of social/lifestyle activities, predicted occurrence of depressive symptoms and of mood symptoms in PwMS younger than 65 years of age. In addition, perceived physical impact of MS on health predicted the occurrence of mood symptoms.

Previously it has been reported that in PwMS the scores of depressive symptoms are stable over time but that the scores of mood symptoms vary [8], a finding which has been confirmed in our previous study of the present sample [13]. However, when using cut-offs to categorise the scores into two groups with regard to the presence or absence of depressive symptoms and the presence or absence of mood symptoms, significant variations over 2 years were found for both variables. More than 40 % of the participants had depressive symptoms at one or several occasions over the 2 years, highlighting the importance of longitudinal assessment in order to identify those PwMS in need of psychological interventions [26].

Our study showed that several variables, other than those which are disease-related, predict the occurrence of depressive symptoms and mood symptoms in PwMS. In all models weak coping capacity as assessed with the SOC Scale was an independent predictor for the occurrence of both depressive symptoms and mood symptoms, This highlights a need for useful coping strategies among PwMS to help them manage living with this chronic disease [6]. The fact that weak coping capacity influenced both mood symptoms and the more global indices of depressive symptoms suggests that coping capacity might influence several areas of functioning in PwMS, which has been reported previously [37]. Our results propose that coping capacity can be a factor influencing the development of or persistence of depressive symptoms in PwMS. SOC was originally believed to be stable after the age of 30, but recently that stability has been debated [38] and SOC may thus be a modifiable factor that needs to be considered in the development of appropriate interventions for PwMS with depressive symptoms. Our results suggest that it might be important for health care services to be able to identify PwMS with a weak coping capacity in order to decrease their risk of developing depressive symptoms and mood symptoms.

Reduced frequency of social/lifestyle activities predicted the occurrence of depressive symptoms as well as mood symptoms in the models where all PwMS were included. Our results highlight that the ability of PwMS to continue their participation in valued everyday activities is an important goal for health services which work with promoting health in this group. However, in the models which only included PwMS of working age, not working rather than having a reduced frequency of social/lifestyle activities was an independent predictor for the occurrence of depressive symptoms and mood symptoms. The ability to work seems to be very important for people of working age for how they can participate in society [39]. This might also positively influence the individual’s economic potential [40]; furthermore, the ability to work is highly esteemed in society. These possible consequences may explain why work status in the present study was a stronger predictor than the more general variable frequency of social/lifestyle activities among PwMS of working age. The meaning PwMS give to their ability to work and how society can best support their decisions and potential regarding participation in the work force need to be more thoroughly explored.

In all GEE models a high psychological impact of MS on health in interaction with time predicted the occurrence of depressive symptoms and mood symptoms. The association found between the psychological impact of MS as assessed with the MSIS-29, and depressive symptoms has been reported previously (27) and our results suggest that the association is also valid in a longitudinal perspective. In Sweden, PwMS are routinely assessed with the MSIS-29 since this instrument is included in the Swedish MS registry and it includes both the psychological and physical impact of MS. Our results indicate that it may be used as a screening instrument to identify those PwMS who need to be assessed with a recommended instrument for detecting depressive symptoms, such as the BDI.

Physical impact of MS on health was a predictor for mood symptoms in PwMS < 65 years of age which might reflect the need for physical capacity from the working environment. Physical as well as psychological barriers in the work environment should be identified and considered when planning for future work-related rehabilitation interventions.

In this study neither fatigue nor cognitive impairment were found to be predictors for the occurrence of depressive symptoms or mood symptoms. Our results differ from those of previous cross-sectional studies implying that other independent variables included in the present analyses might have had a stronger predictive capacity than fatigue or cognitive impairment [14, 15]. Thus, our results need to be confirmed in further studies. When interpreting our results one should also keep in mind that every third PwMS received anti-depressant treatment over a shorter or longer timeframe during the 2 years and that the majority of the sample received immunomodulatory treatment, which might have influenced the results.

## Conclusions

The occurrence of depressive symptoms and mood symptoms in PwMS vary over a 2 year period, and almost half of the PwMS have depressive symptoms at least once during that period. Health care services should develop strategies aimed at identifying PwMS who are depressed or who develop depressive symptoms over time. Interventions for alleviating depressive symptoms should take into account the individual’s coping capacity and their perceived impact of MS on health, and facilitate their ability to maintain participation in valued everyday activities.

## Abbreviations

EDSS: expanded disability status scale
FAI: Frenchay Activities Index
FSS: Fatigue Severity Scale
GEE: Generalised Estimating Equations
MS: multiple sclerosis
MSIS-29: Multiple Sclerosis Impact Scale
PwMS: people with multiple sclerosis
SDMT: Symbol Digit Modalities Test
SOC: sense of coherence
